# Unveiling the therapeutic potential of gut microbiota metabolites for the treatment of renal fibrosis based on network pharmacology study

**DOI:** 10.1186/s40643-026-01083-8

**Published:** 2026-06-20

**Authors:** Weiguo Yao, Jinlin Huo, Kun Liu, Pengyu Tao

**Affiliations:** 1 Department of Nephrology, Jinshan District Central Hospital Affiliated to Shanghai University of Medicine and Health Sciences, Shanghai, China; 2 Clinical Medical Research Center, The First Affiliated Hospital of Shantou University Medical College, Shantou, China

**Keywords:** Gut microbiota, Metabolites, Renal fibrosis, Bioinformatics analysis, Network pharmacology

## Abstract

**Background:**

Renal fibrosis is a progressive injury contributing to renal function deterioration. Mounting evidence has underscored the profound impact of gut microbiota metabolites on host health and disease, yet their underlying mechanisms against renal fibrosis remain unclear. The aim of this study was to fully elucidate their therapeutic potential in the context of renal fibrosis.

**Methods:**

The targets of gut microbiota metabolites were identified in gutMGene. The diseases targets were obtained from the OMIM, GeneCards and DisGeNet databases. The STRING and DAVID platform were employed to identify the core targets and pathways. Gut Microbiota-Targets-Pathway-Metabolites (G-T-P-M) network was constructed to screen the core metabolites. Molecular docking was used to assess the interactions between the targets and metabolites.

**Results:**

A total of 47 overlapping targets related to gut microbiota metabolites and renal fibrosis were acquired. The bioinformatics analysis indicated that the targets were enriched in the regulation of TNF pathway and Toll-like receptor pathway. The PPI network (Protein-Protein Interaction) identified JUN, IL6, IL1B and AKT1 as the core targets. The G-T-P-M network revealed that Propionate, Butyrate and 3-Indolepropionic acid were identified as the core non-toxic and promising core metabolites. The core metabolites showed stable binding affinity with the core targets.

**Conclusion:**

The findings highlight that gut microbiota metabolites represent a promising therapeutic option for combating renal fibrosis by modulating multiple targets and pathways, providing a theoretical foundation for the future studies exploring gut microbiota as targeted strategies in the prevention and treatment of renal fibrosis.

**Graphical abstract:**

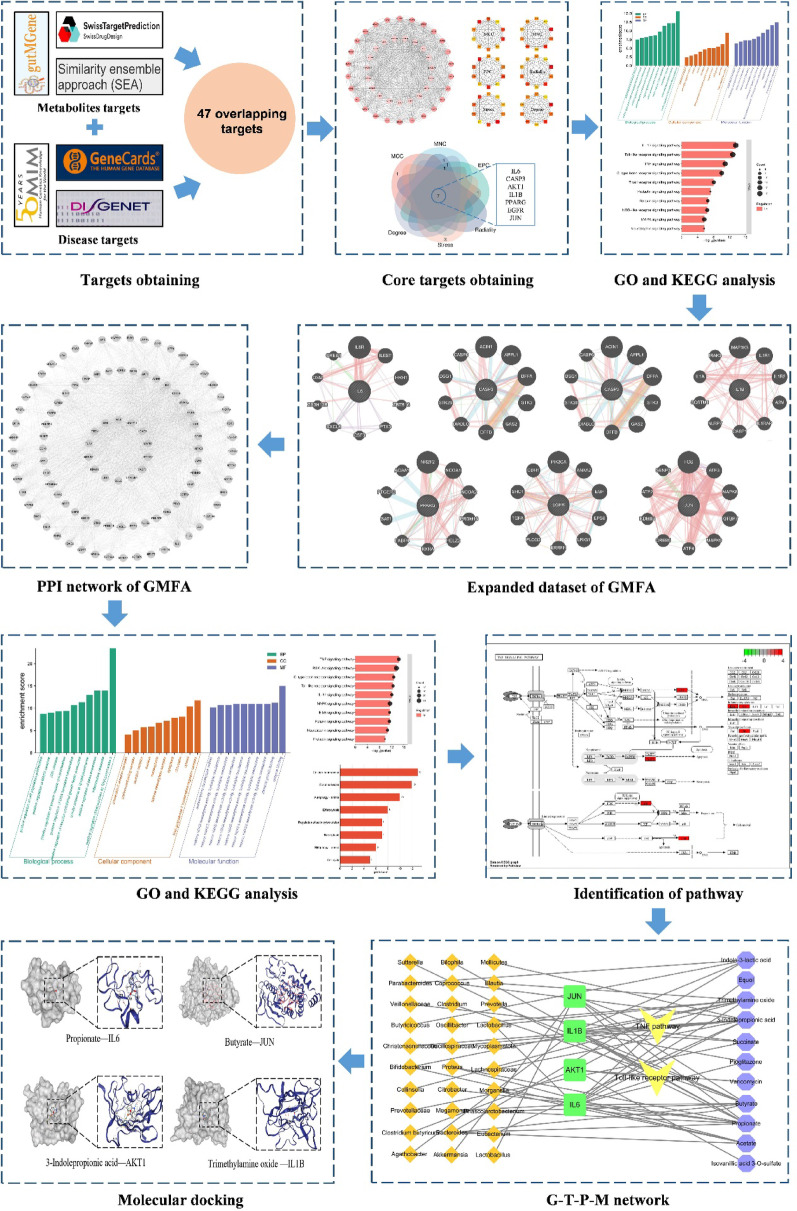

## Introduction

Renal fibrosis, a pivotal pathological feature in chronic kidney diseases (CKD), is marked by the abnormal deposition of extracellular matrix, tubular atrophy, and mitochondrial dysfunction (Abbad et al. 2025). The mechanisms of renal fibrosis involve multiple aspects: oxidative stress, abnormal activation of inflammatory signaling pathways, telomere shortening, and DNA damage-induced tubular epithelial cell senescence (Liu 2024, Miao et al. 2024). The pathological damage mediated by these complex mechanisms is considered the leading contributor to tubular injury and glomerulosclerosis, as it triggers inflammatory responses, myofibroblast activation, and macrophage infiltration that lead to the loss of renal function (Miao et al. 2024, Cao et al. 2022, Liu et al. 2024). The continuous progression of renal fibrosis may culminate in end-stage renal disease (ESRD), which is regarded as a significant risk factor for high morbidity and mortality in CKD (Song et al. 2024). Current therapeutic approaches for renal fibrosis have failed to yield satisfactory results. Thus, there is an urgent need to develop cost-effective strategies to delay or reverse the adverse complications of renal fibrosis.

Recently, amounting evidence has suggested that the metabolites derived from gut microbiota could significantly impact disease onset, including obesity, diabetes, and kidney diseases (Chen et al. 2025). Dysbiosis triggers the production of harmful metabolites like Indoxyl sulfate and Trimethylamine-N-oxide, leading to epithelial-to-mesenchymal transition (EMT) and extracellular matrix deposition by inducing oxidative stress and inflammation (Zhou et al. 2024). Conversely, short-chain fatty acids (SCFAs) like acetate, propionate and butyrate produced by beneficial bacteria could enhance intestinal barrier integrity, reduce bacterial translocation, and mitigate inflammation via histone deacetylase inhibition and G protein-coupled receptor activation (Portincasa et al. 2022, Tang et al. 2022, Fusco et al. 2023). It has been reported that lower levels of SCFAs were found in CKD patients compared to the healthy individuals (He et al. 2024), while the exogenous SCFAs supplementation could improve renal dysfunction in animal experiments by alleviating inflammation and reducing collagen synthesis (Tian et al. 2023). These protective effects are associated with the beneficial metabolites’ capacity to prevent the uremic toxins and pathogens from entering the bloodstream by maintaining barrier integrity (Yuan et al. 2024). Thus, it is evident that the gut-kidney axis’ bidirectional regulation could be employed as a therapeutic strategy to prevent or delay renal fibrosis.

**Fig. 1:**
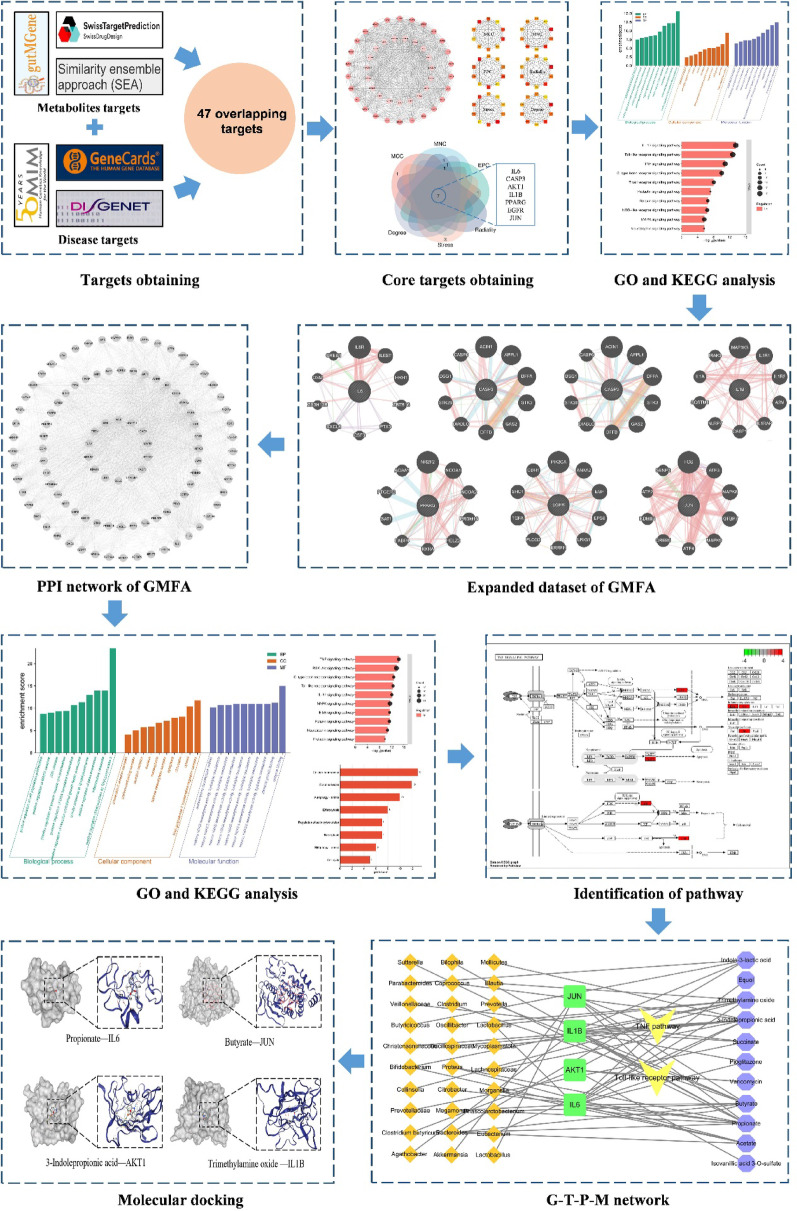
The flowchart of the study design. First, targets of gut microbiota metabolites were predicted using the SEA and STP. Second, renal fibrosis-related targets were retrieved from GeneCards, OMIM, and DisGeNET databases. The overlapping targets were identified by VENN diagram. KEGG and GO enrichment analysis were performed on the overlapping targets. PPI network used to screen core targets. The GeneMANIA functional association network was constructed to expand target dataset. Finally, the Gut Microbiota–Targets–Pathways–Metabolites (G-T-P-M) network was established to screen core metabolites

An emerging computer-based network analytical approach involves the combination of multiple technologies such as bioinformatics enrichment analysis, protein–protein interaction (PPI) network analysis, and even topological analysis (Zhang et al. 2024). This novel analytical approach could serve as a paradigm to decode the molecular and biological information attributing to complex diseases. Compared to traditional analytical methods, network pharmacology has changed the research pattern from “one target, one compound” to “multiple targets, multiple compounds” (Yao et al. 2025). By constructing the network of compound–target–pathway-disease, it helps to elucidate the therapeutic values of these interactions contributing to the management of diseases. Due to the properties of simple operation and accurate results, this novel analytical approach could be used in a variety of fields, including environmental health, food safety, pharmaceutical research, and disease control. Therefore, we applied computer-based network analytical approach to investigate the beneficial effects of gut microbiota metabolites on alleviating renal fibrosis in the present study. The study is compliant with the TITAN Guidelines (Premier Science et al. 2025). The flowchart of this study was described in Fig. 1.

## Methods and materials

### Data source and tool

The tools used for data collection and visualization was available in Table 1.

**Table 1:** Database, software and analysis platform

No	Database and analysis platform	Source	Version
1	gutMGene	http://bio-annotation.cn/gutmgene/	\
2	Swiss target prediction	http://www.swisstargetprediction.ch/	\
3	Pubchem	https://pubchem.ncbi.nlm.nih.gov/	\
4	bioinformatics	http://www.bioinformatics.com.cn/	\
5	STRING	https://string-db.org	V12.0
6	Cytoscape software	https://cytoscape.org/	V3.7.1
7	OMIM	https://omim.org/	\
8	Genecards	https://www.genecards.org/	\
9	DisgeNet	https://www.disgenet.org/	\
10	DAVID bioinformatics	https://david.ncifcrf.gov/tools.jsp	\
11	Similarity ensemble approach	https://sea.bkslab.org/	\
12	SwissAMDE	http://www.swissadme.ch/index.php	\
13	ADMETlab	https://admetmesh.scbdd.com/	\

### The collection of targets

The metabolites of gut microbiota were retrieved from the gutMGene. Their SMILES notations were then downloaded from PubChem and uploaded to two distinct target prediction tools: Similarity Ensemble Approach (SEA) and Swiss Target Prediction (STP). The intersection of predicted targets from these two platform was defined as the collective targets of gut microbiota metabolites and Peach kernel.

### The identification of potential targets of renal fibrosis

The keyword “renal fibrosis” was searched the known renal fibrosis related targets in GeneCards and DisGeNET database. The targets associated with renal fibrosis of the two database were merged after discarding the repeated targets.

### The identification of core targets and bioinformatics analysis

The protein-protein interaction (PPI) network was constructed by uploading the common targets to the STRING platform. The CytoHubba plugin within the cytoscape software was employed to identify crucial targets within this network leveraging multiple analytical methods, including the MCC, MNC and Degree. The intersection targets of these three mthods were ultimately recognized as the core targets. The common targets were uploaded to the DAVID platform to perform GO and KEGG functional analysis. The GO analysis encompasses three aspects: BP (Biological Process), CC (Cellular Component) and MF (Molecular Function), and we selected the top 10 terms from GO and KEGG for visualization based on *P* value < 0.05.

### Molecular docking verification

Structural data for components and targets were retrieved from the Protein Data Bank and PubChem database respectively. The target protein and ligand were isolated using PyMOL software. AutoDock Tools was employed to generate pdbqt formats for target proteins, ligands, and constituents. Subsequently, the receptor’s active pocket was constructed via the Grid plugin. Ultimately, Vina was utilized to calculate the binding energy between the target and component. To form a stable structure between the receptor and ligand, their binding energy is typically less than 0 kcal·mol^− 1^. Generally speaking, binding energy < − 5.0 kcal/mol indicated moderate affinity and < − 7.0 kcal/mol indicated strong affinity.

## Results

### The identifications of targets related to gut microbiota metabolites and renal fibrosis

**Fig. 2:**
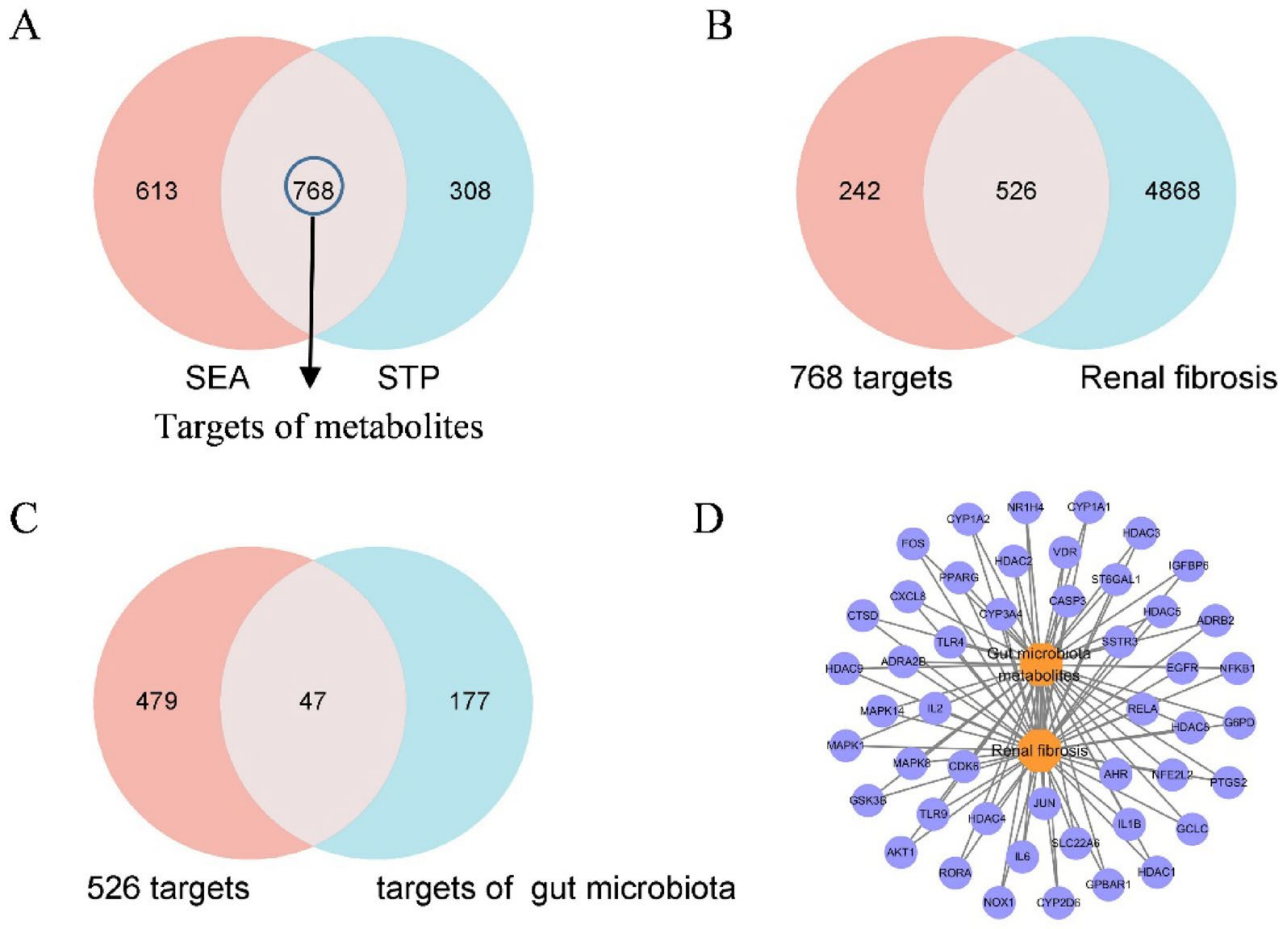
The potential targets acquisition. **A** The overlapping targets of gut microbiota metabolites between SEA and STP. **B** The overlapping targets between disease targets and gut microbiota metabolites. **C** The overlapping targets between 526 targets and gut microbiota targets. **D** Visualized network illustrating the complex relationships among gut microbiota metabolites, renal fibrosis and the 47 targets

As shown in Fig. 2A, after searching the SEA and STP databases, we obtained 1381 and 1075 targets related to gut microbiota metabolites, respectively. The 768 targets that appeared in the Venn diagram were considered the overlapping targets between SEA and STP. A total of 5394 renal fibrosis targets were acquired after consolidation and de-duplication (Fig. 2B). Subsequently, the 526 overlapping targets in the Venn diagram were the hub targets between gut microbiota metabolites and renal fibrosis (Fig. 2B). Lastly, by intersecting the 526 targets with 224 gut microbiota targets, we obtained a total of 47 overlapping targets, which were regarded as the pivotal targets involved in the regulation of renal fibrosis (Fig. 2C). We also built a metabolites-target-disease network to visualize their complex relationships (Fig. 2D).

### The prediction of core targets in PPI network

**Fig. 3:**
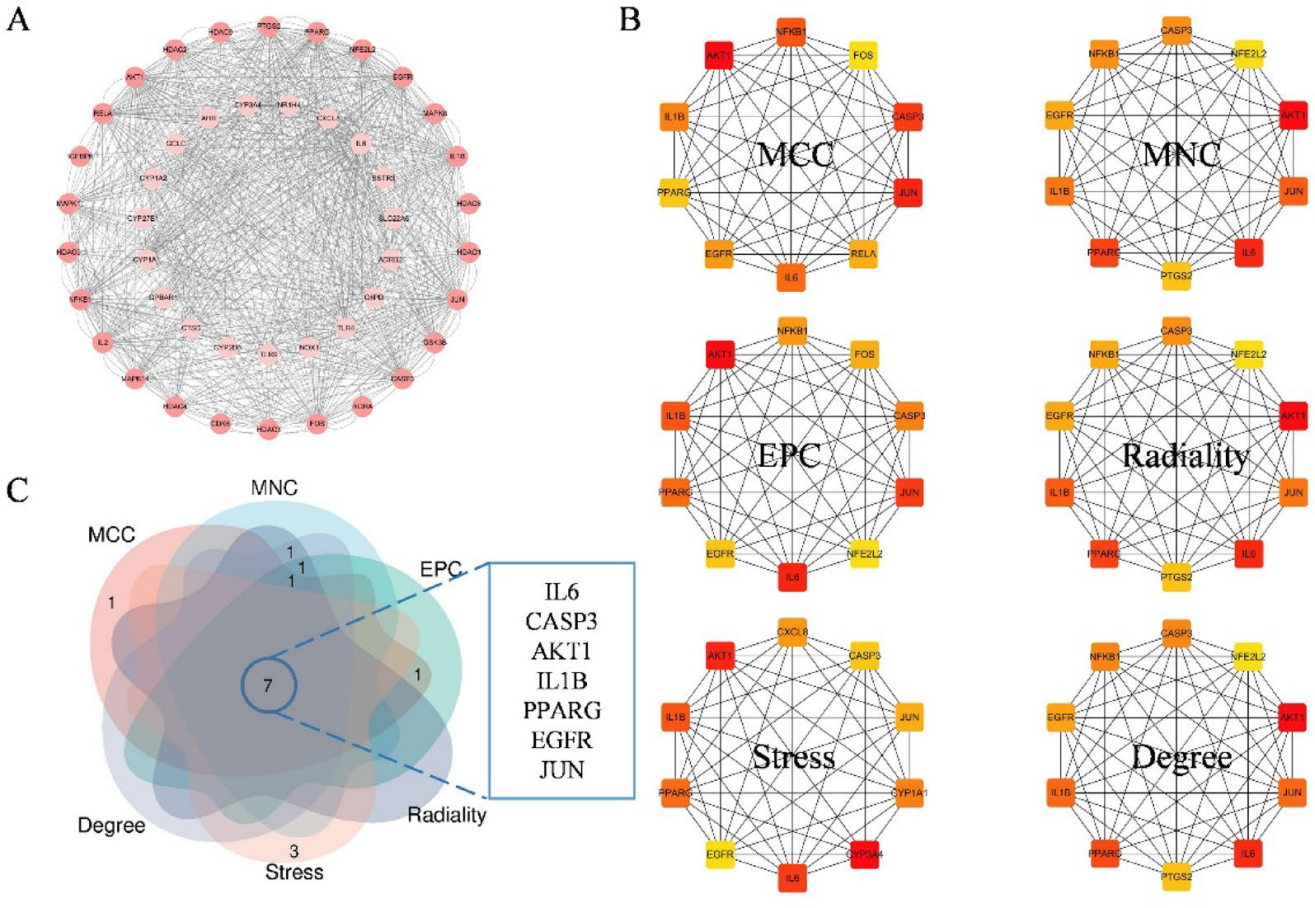
The identification of core targets. **A** The PPI network of overlapping targets. **B** Top 10 core targets ranked by six topological algorithms (MCC, MNC, EPC, Stress, Degree, Radiality) in CytoHubba plugin. **C** The 7 core targets acquired from 6 algorithms using VENN diagram

In order to filter out the core therapeutic targets against renal fibrosis, PPI network consisting of 45 nodes and 734 edges was successfully constructed (Fig. 3A). To further screen core targets, we applied six algorithms from the CytoHubba plugin in Cytoscape 3.6, including MCC, MNC, EPC, Stress, Degree and Radiality to further filter the core targets from PPI network. As depicted in Fig. 3B, the top 10 targets from each algorithm were selected, and their intersection targets were identified using a VENN diagram. The results revealed that a total of seven genes were considered core therapeutic targets for renal fibrosis treatment, namely, IL6, AKT1, CASP3, IL1B, PPARG, EGFR and JUN (Fig. 3C).

### The GO and KEGG enrichment analysis

**Fig. 4:**
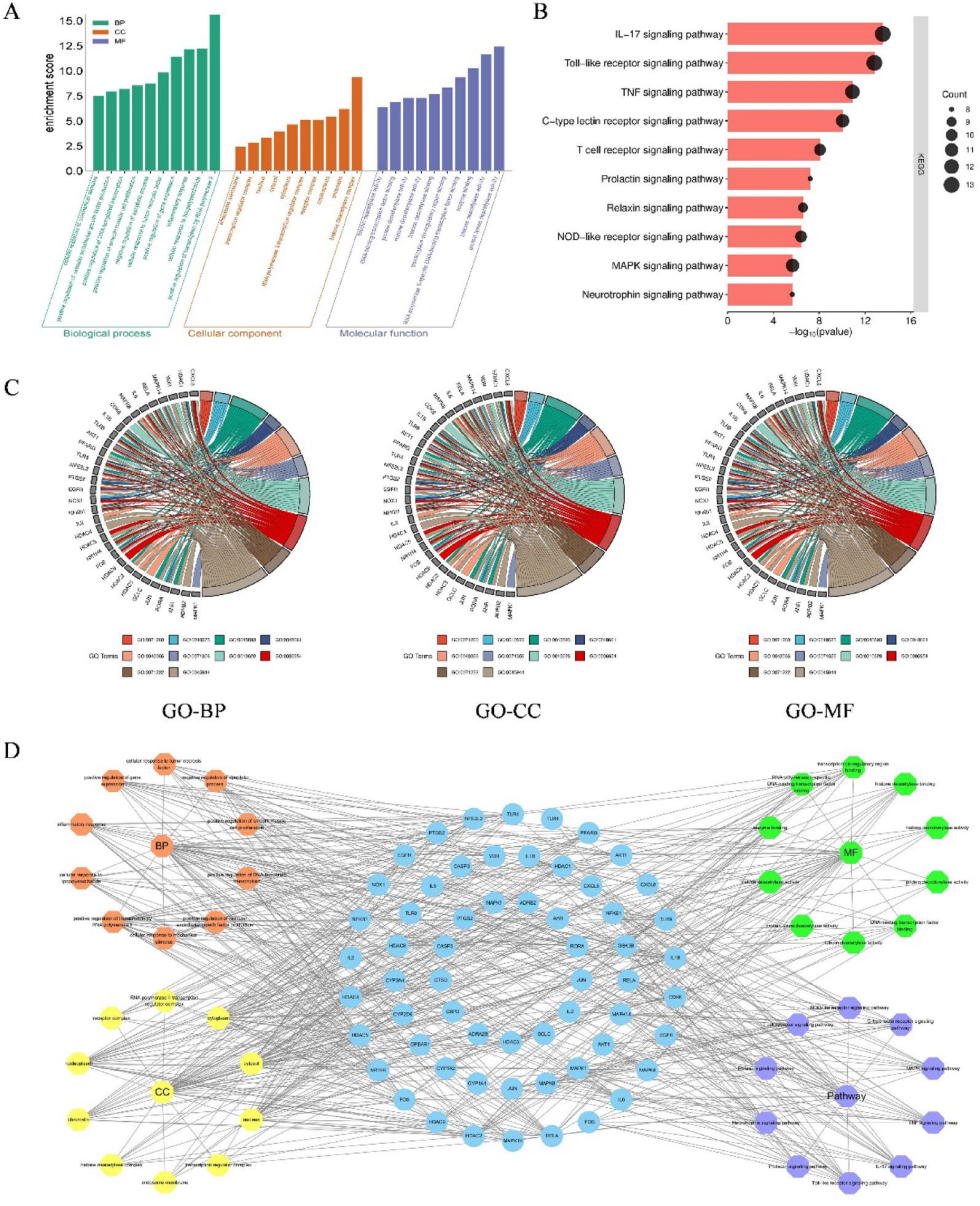
Biological analysis of gut microbiota metabolites against renal fibrosis. **A** Top 10 significantly enriched terms in biological process (BP), cellular component (CC), and molecular function (MF). **B** The top 10 KEGG pathways based on *P* value. **C** The genes involved in the regulation of BP, CC and MF. **D** The BP-CC-MF-Target-Pathway network. Blue nodes: targets, brown nodes: BP, yellow nodes: CC, green nodes: MF, purple nodes: signaling pathways

In order to get a better knowledge of gut microbiota metabolites against renal fibrosis, we conducted GO function and KEGG enrichment analysis on DAVID platform. For GO enrichment analysis, we selected the top 10 terms from BP, CC and MF based on the threshold value of FDR that was set at < 0.05. As shown in Fig. 4A, most of the terms are enriched in mechanical stimulus, tumor necrosis factor, apoptotic process, vascular endothelial growth factor production and smooth muscle cell proliferation. Notably, the genes were mainly located in nucleus, cytosol and cytoplasm. We also observed that the MF of the genes were closely related to histone decrotonylase activity, deacetylase activity and enzyme binding. The Fig. 4C revealed the targets involved in the regulation of BP, CC and MF. The KEGG enrichment results revealed the pathways related to our input gene set including Toll-like receptor signaling pathway, IL-17 signaling pathway, TNF signaling pathway, C-type lectin receptor signaling pathway, NOD-like receptor signaling pathway, and more (Fig. 4B). We also constructed a network containing BPs-CCs-MFs-Targets-Pathways to display their mutual relationships, where the blue nodes signified targets, brown nodes signified BP, yellow nodes signified CC, green nodes signified MF and the purple nodes signified pathways (Fig. 4E). These findings indicated that gut microbiota metabolites exerted their therapeutic functions by regulating multi-biological functions as wells as multi-pathways.

### GeneMANIA functional association (GMFA) network analysis of seven core targets

**Fig. 5:**
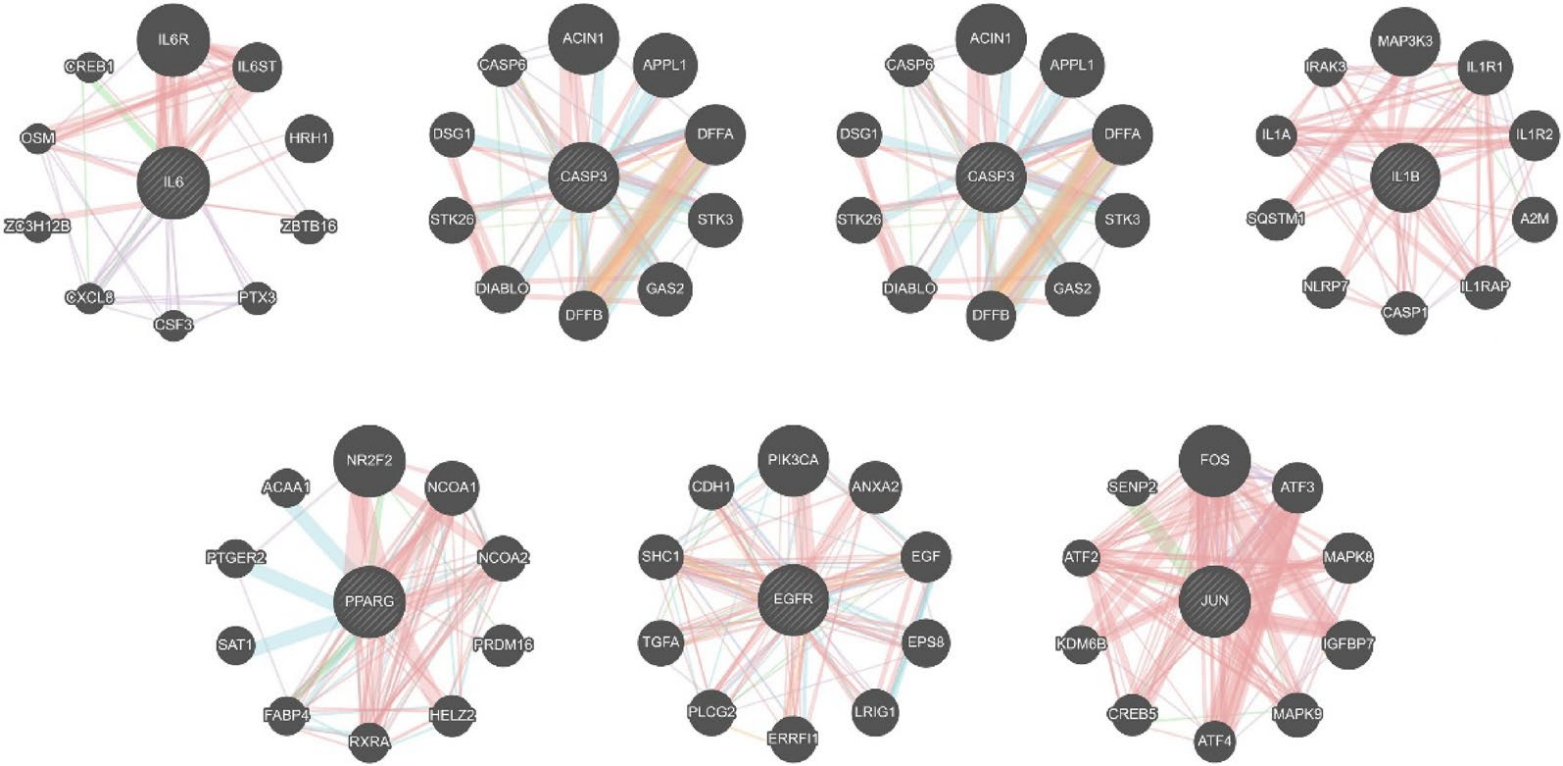
Expanded target dataset generated by GeneMANIA based on seven initial core targets. The network shows co-expression, genetic interaction, and physical association patterns; each core target was used to predict top 10 functionally related genes, generating an expanded dataset for further analysis

GeneMANIA serves as powerful tools for exploring functional associations. By analyzing co-expression patterns, genetic interactions and physical associations among the input genes, this novel approach enables the users to predict additional genes linked to a specified input gene, many of which could emerge as highly valuable therapeutic targets. Based on the advantage of GeneMANIA, we expanded the data set of the eight core genes by adding additional top 10 genes for each input gene (Fig. 5). Building upon this progression, we integrated these newly identified genes with the initial 47 targets, culminating in a refined dataset encompassing 106 targets. Through this process, we could more accurately highlighted the genes that hold great potential as therapeutic targets.

### The functional analysis of GMFA network

**Fig. 6:**
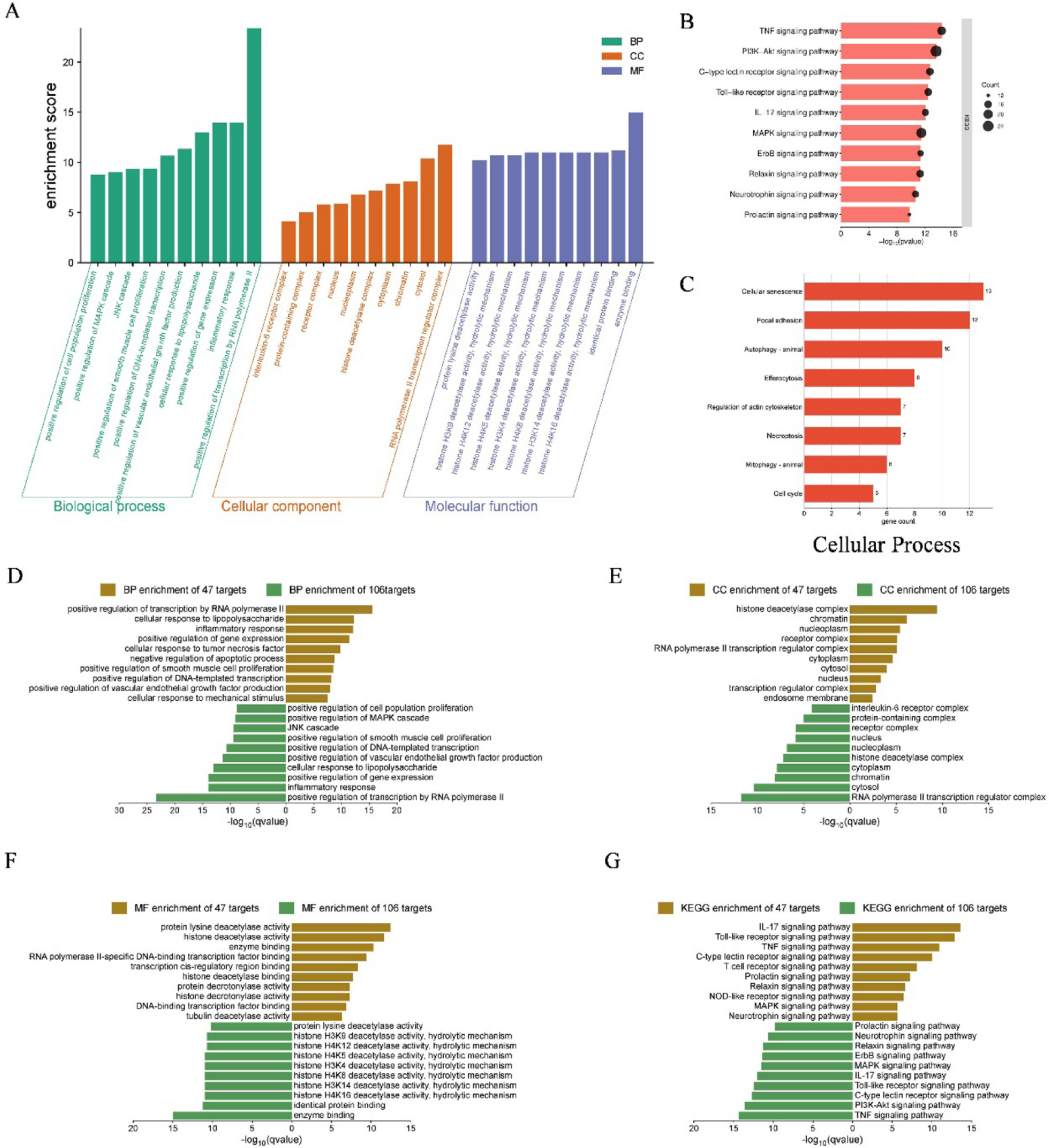
GO and KEGG enrichment analysis of the expanded core target dataset. **A** Top 10 enriched BP, CC, and MF terms of the 106 expanded targets. **B** Top 10 enriched KEGG signaling pathways of the 106 expanded targets. **C** Top 10 cellular process, including cellular senescence, focal adhesion, autophagy, and necroptosis. **D**, **E **and **F** The comparison of BP, CC and MF of 47 hub targets and expanded target dataset. **G** The comparison of KEGG enrichment analysis of 47 hub targets and expanded target dataset

**Fig. 7:**
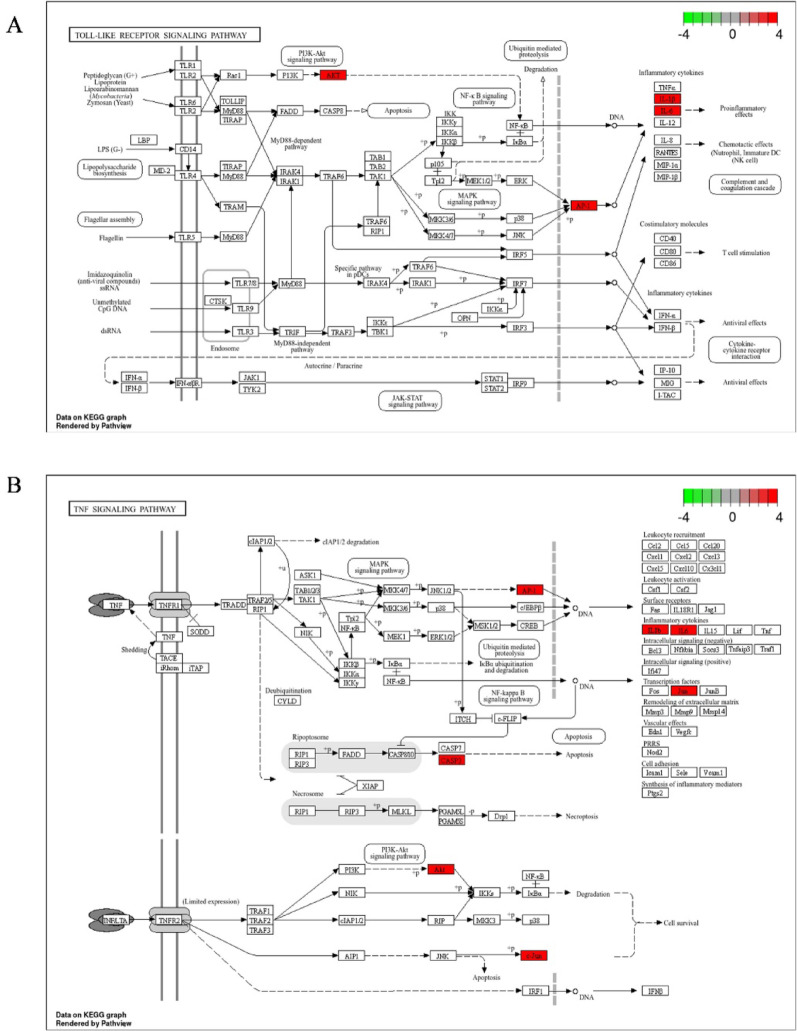
Identification of core signaling pathways against renal fibrosis. **A** Key genes involved in the Toll-like receptor signaling pathway. **B** Key genes involved in the TNF pathway. Core targets JUN, IL1B, AKT1, and IL6 are highlighted in red

To further gain a deep insight into the function of 106 targets derived from the GMFA data set, we carried out a comprehensive analysis of GO and KEGG enrichment analysis of the 106 targets. As shown in Fig. 6A, the BP of the GMFA data were mostly related to regulation of cell population proliferation, MAPK cascade, gene expression, inflammatory response, and more. The CC of the GMFA data were mostly located in nucleus, nucleoplasm, histone deacetylase complex, cytoplasm, and more. The MF of the genes were closely related to histone deacetylase activity and enzyme binding. As shown in Fig. 6B, the top 10 pathways were IL-17 signaling pathway, Toll-like receptor signaling pathway, C-type lectin receptor signaling pathway, PI3K-Akt signaling pathway, TNF signaling pathway, and more. These pathways also governed the cellular function, such as cellular senescence, focal adhesion, autophagy, necroptosis, and more (Fig. 6C). Furthermore, we obtained the two key pathways from the two KEGG enrichment analysis, namely Toll-like receptor signaling pathway and TNF signaling pathway (Fig. 7A–B). The core targets implicated in both pathways are highlighted in red, specifically JUN, IL1B, AKT1 and IL6, underscoring their central roles in the underlying molecular mechanisms. A visual comparison of the GO and KEGG enrichment outcomes was made between the 106 targets and 47 targets (Fig. 6D–G). Overall, the analyses of 106 targets yielded more comprehensive and detailed insights into their roles in regulating renal fibrosis, illuminating the intricate molecular mechanisms underlying the effects of gut microbiota metabolites.

### Functional clustering analysis

**Fig. 8:**
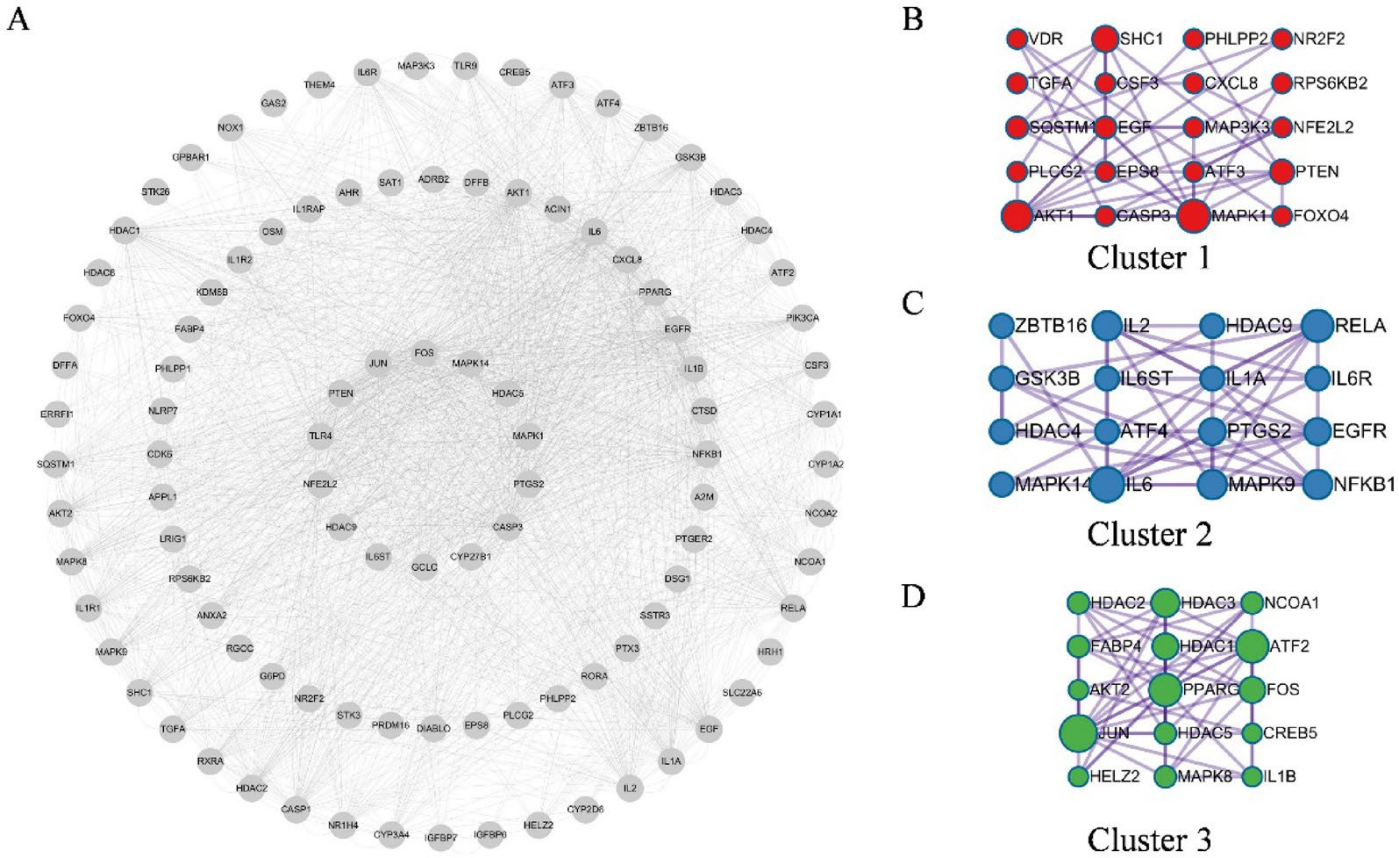
The cluster of GMFAN. **A** The PPI network of expanded core target dataset. **B**, **C** and **D** Three key functional modules (Cluster 1, Cluster 2, Cluster 3) identified by MCODE clustering analysis

**Fig. 9:**
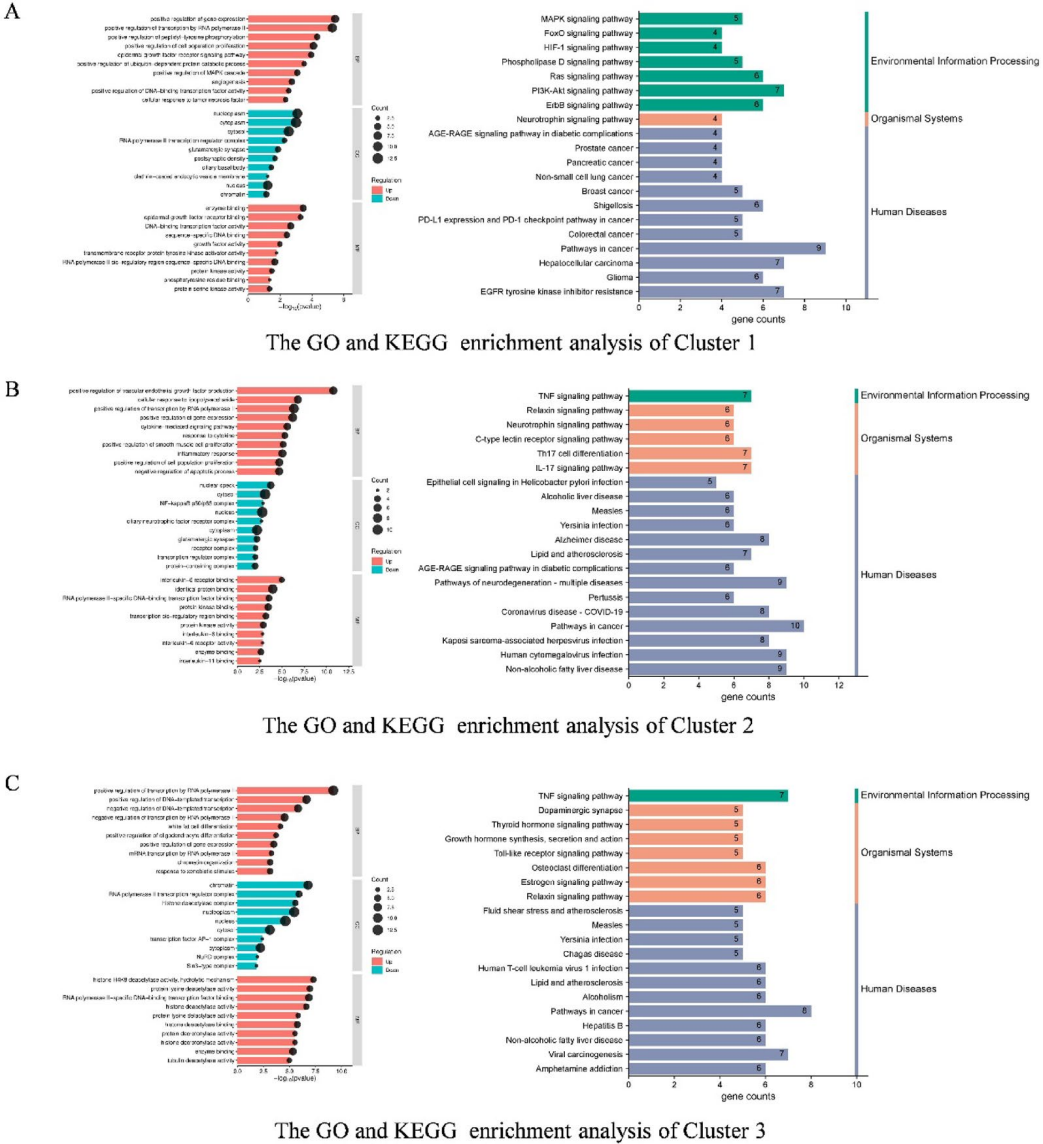
The enrichment analysis of three key functional modules. **A** The GO and KEGG enrichment analysis of Cluster (1) **B** The GO and KEGG enrichment analysis of Cluster (2) **C** The GO and KEGG enrichment analysis of Cluster 3

The 106 targets were submitted to STRING platform to construct a PPI network which could help to understand the interactions among all the targets (Fig. 8A). To further acquire more information about the network, Metascape was used for MCODE analysis. As shown in Fig. 8B–D, the network could be divided into 3 cluster. In addition, we conducted GO and KEGG enrichment analysis on Cluster 1, 2 and 3. As shown in Fig. 9A–C, the results revealed that Cluster 1, 2 and 3 shared the similar GO terms such as regulation of transcription by RNA polymerase II, positive regulation of gene expression, cellular response to tumor necrosis factor, cytosol, cytoplasm, nucleoplasm, protein serine kinase activity, phosphotyrosine residue binding, enzyme binding and more. Furthermore, the KEGG enrichment analysis of the three clusters could be categorized into multiple distinct groups. These categories encompass aspects related to environmental information processing, cellular processes, organismal systems, and diseases. Notably, these pathways covered a wide range of human diseases, including Hepatitis, Non-alcoholic fatty liver disease, Alzheimer disease, Measles, and more. This comprehensive clustering analysis elucidates the intricate mechanisms of metabolites, including the underlying biological processes, molecular functions, and potential therapeutic cellular signaling pathways. By unraveling these complex interactions, the study provides invaluable insights that not only deepen our understanding of biological systems but also serve as a foundation for future research.

### Identification of core metabolites and molecular docking

**Fig. 10:**
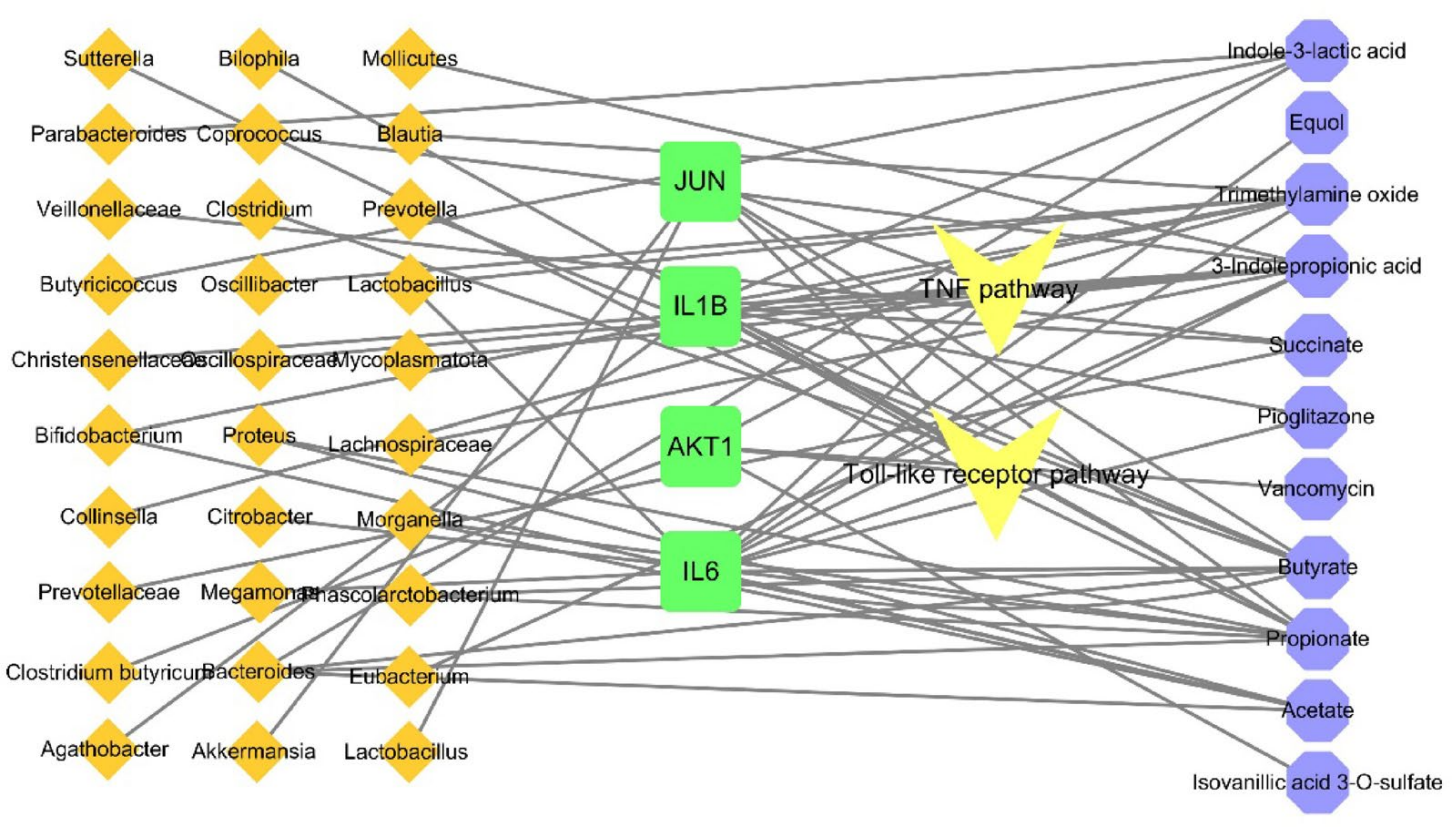
The gut microbiota-targets-pathways-metabolites network. The network showing relationships among gut microbiota (brown nodes), core targets (green nodes), key pathways (yellow nodes), and metabolites (purple nodes). The network includes 30 gut microbiota, 4 core targets, 2 key pathways, and 9 metabolites

**Fig. 11:**
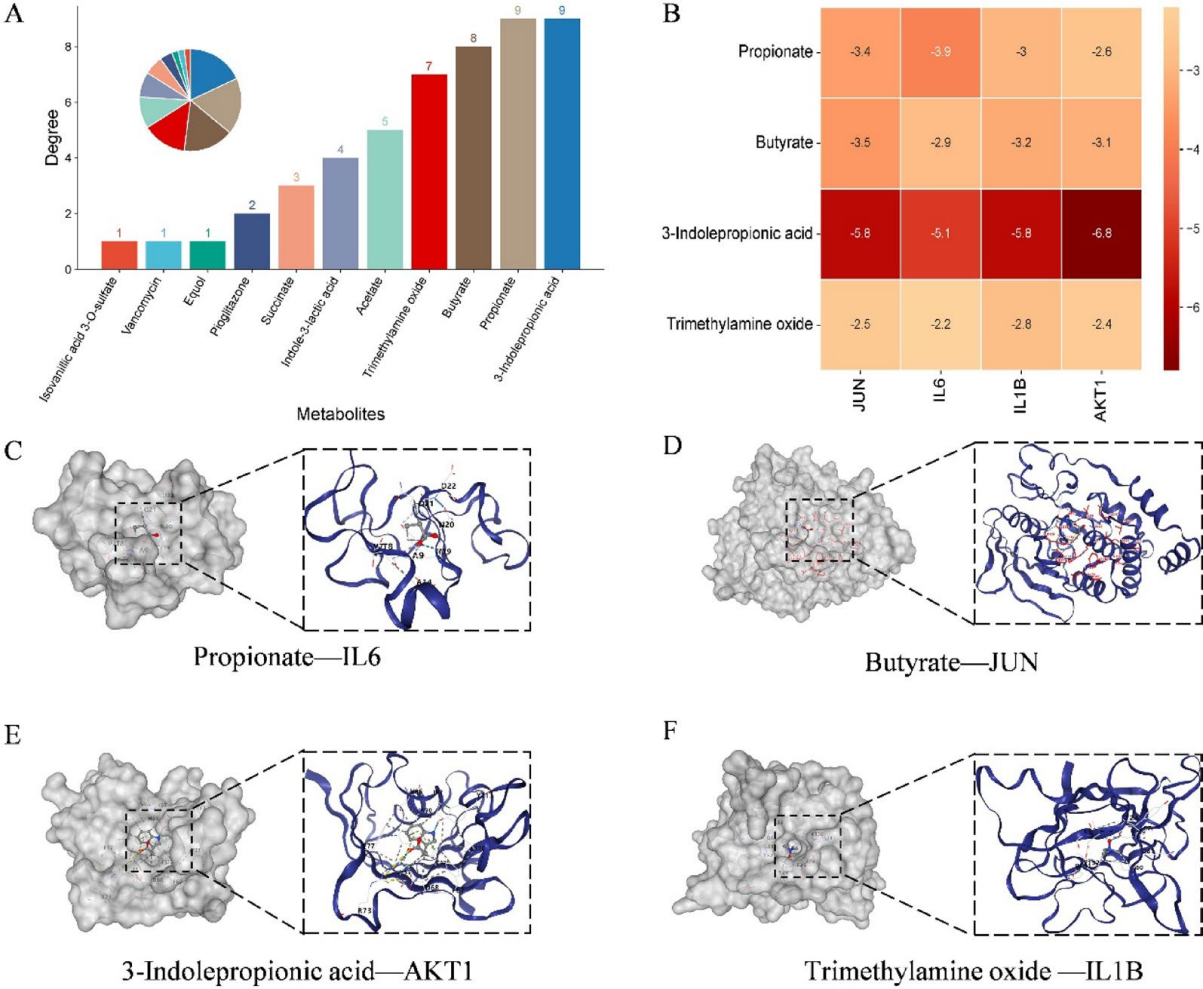
Molecular docking results. **A** The identification of top 4 metabolites. **B** The heatmap reflecting the binding energy between metabolites and targets. **C** Molecular docking of propionate with IL6. **D** Molecular docking of butylate with JUN. **E** Molecular docking of 3-Indolepropionic acid with AKT1. **F** Molecular docking of Trimethylamine oxide with IL1B

The associations among Gut Microbiota-Targets-Pathway-Metabolites (G-T-P-M) was visualized by constructing (G-T-P-M) network. In the network, the blown nodes signified the gut microbiota, the green nodes signified target, the yellow nodes signified pathway and the purple nodes signified gut microbiota metabolites (Fig. 10). The G-T-P-M network consisted of four targets, thirty gut microbiota, two pathways and nine gut microbiota metabolites. Notably, IL1B and IL6 exhibited the highest number of connections with metabolites, while butyrate and 3-indolepropionic acid showed the most extensive associations with gut microbiota, suggesting that these metabolites and targets might contribute to the regulation of renal fibrosis. What’s more, we filtered out the core gut microbiota metabolites in this G-T-P-M network using Network analyzer tool. The results revealed that Propionate, Butyrate, 3-Indolepropionic acid and Trimethylamine oxide were recognized as the core metabolites after analyzing their Degree score in the network (Fig. 11A).

The molecular docking is an important tool in the network pharmacological study. The application of Molecular docking can predict the affinity between receptor and ligand (such as binding energy, hydrogen bond interactions, etc.) as well as validate the possibility of component-target interactions. All docking scores in our study were negative (< 0 kcal/mol), confirming the metabolites and core targets could form **s**table complexes (Fig. 11B). Notably, 3-Indolepropionic acid exhibited moderate–strong binding to AKT1 (− 6.8 kcal/mol), while the Propionate-IL6 (− 3.9 kcal·mol^− 1^), Butyrate-JUN (− 3.5 kcal mol^− 1^) and Trimethylamine oxide-IL1B (− 2.8 kcal·mol^− 1^) showed weak moderate stable binding. In addition, these results were used for molecular docking visualizations (Fig. 11C–F). Overall, this notable finding underscores the strong interaction between these metabolites and targets, suggesting that they may exert significant regulatory effects through these binding events.

## Discussion

Renal fibrosis is a leading cause of renal failure (Liu et al. 2026). Currently, treatment options for renal fibrosis are limited. Hence, seeking an effective solution to attenuate the health issues caused by renal fibrosis holding important clinical significance. The application of gut microbiota metabolites treatment has made a hot topic of research in recent times (Rong et al. 2024). Network pharmacology, as a widely used advanced methodology, can not only predict the potential targets or pathways involved in the complex biological system of diseases but also identifies the potential drugs targeting diseases intervention (Zhao et al. 2023). In this study, we take the advantage of network pharmacology and molecular docking to investigate the potential mechanisms of gut microbiota metabolites against renal fibrosis. This novel approach unveils that some crucial signaling pathways such as Toll-like receptor signaling pathway and TNF signaling pathway were involved in the development of renal fibrosis. The core targets-JUN, IL1B, AKT1 and IL6 are well-established regulators of renal fibrosis, and their interaction with core microbiota metabolites like Propionate, Butyrate, 3-Indolepropionic acid and Trimethylamine oxide provides deep insight into the metabolites’ mechanisms against renal fibrosis.

In our study, we successfully pinpointed four core therapeutic targets of renal fibrosis that hold potentials for the treatment of renal fibrosis based on comprehensive PPI network analysis. AKT1, also known as protein kinase B, serves as a pivotal regulatory kinase in transducing signals that govern cell growth and survival (Zhang et al. 2024). In the kidney, abnormal activation of AKT1 is closely associated with the occurence renal fibrosis. AKT1 activation promotes the TGF-β/Smad signaling pathway, inducing tubular epithelial cells to express α-smooth muscle actin (α-SMA) and fibronectin while inhibiting E-cadherin (Jere et al. 2022). This process drives the epithelial-mesenchymal transition (EMT) process, prompting epithelial cells to transform into myofibroblasts and exacerbating extracellular matrix (ECM) deposition (Wang et al. 2023). In addition, AKT1 amplifies the inflammatory cascade in the kidney by activating the NF-κB pathway, promoting the secretion of pro-inflammatory cytokines such as IL-6 and TNF-α (Pan et al. 2023). This process recruits macrophages and lymphocytes to infiltrate renal tissues that exacerbate inflammatory injury. Interleukin-6 (IL-6) acts a vital regulator in the pathogenesis of various kidney diseases, acting as a key mediator of inflammation and tissue injury (Takagaki et al. 2020). The elevated IL-6 levels were correlated with the severity of chronic kidney disease (CKD) that led to fibrosis, endothelial dysfunction, and immune dysregulation (Batra et al. 2021). Under the condition of diabetic nephropathy (DN), the upregulation of IL-6 levels led to glomerular hypertrophy, ECM deposition and mesangial expansion through promoting mesangial cell proliferation and emission of pro-inflammatory cytokine (Guo et al. 2022). Some studies reported that the administrations of gut microbiota metabolites like propionate or butyrate was capable of reducing collagen deposition and downregulating the renal fibrosis markers like Col-I and Col-III through inhibiting the IL-6 mediated inflammation (Liu et al. 2020). The gut microbiota-derived SCFAs promote M2 macrophage polarization via inhibiting AKT/mTOR signaling by reducing the ratio of pro-inflammatory M1 macrophage infiltration in the kidney that contribute to alleviate AKT-mediated fibrotic responses (Stoeva et al. 2021). Therefore, the inhibition of IL-6 or AKT1 by gut microbiota metabolites may represent a novel approach to managing renal fibrosis.

The KEGG enrichment analysis revealed that TLR signaling pathway and TNF signaling pathway were the critical pathway that could influence the development of renal fibrosis. The TLR family consists of 10 evolutionarily conserved members (TLR1 to TLR10), playing a pivotal role in immune system (Sahoo 2020, Kaur et al. 2022). TLR-9 could induce the generation of pro-inflammatory factors and fibrosis-related genes (Saber et al. 2022). In the kidney, TLR-9 is predominantly expressed in renal tubular epithelial cells, macrophages, and other cell types. In a mouse model of ischemia-reperfusion injury (IRI), TLR-9 knockout suppresses the epithelial-mesenchymal transition (EMT) process, characterized by upregulated E-cadherin expression, downregulated α-smooth muscle actin (α-SMA) expression, and reduced mRNA levels of fibrosis-related factors such as COL-I, COL-III and Snail1 (Han et al. 2020). Additionally, pharmacological or genetic inhibition of TLR-9 disrupts M2 macrophage recruitment, mitigates inflammatory signaling cascades, and halts EMT progression, thereby delaying the development of renal fibrosis(Liu and Zen 2021, Huang et al. 2024). Tumor necrosis factor (TNF) pathway plays a significant role in renal fibrosis by modulating macrophage polarization, inflammatory responses and EMT (O’Sullivan et al. 2023). Renal injury triggers the overexpression of TNF that promotes macrophage infiltration and enhance the release of pro-inflammatory mediators, which in turn accelerate renal tissue injury (Swart et al. 2022). What’s more, TNF could induce EMT in renal tissue by downregulating E-cadherin and upregulating α-smooth muscle actin (α-SMA), driving myofibroblast activation and collagen synthesis (Lousa et al. 2022). The pharmacological or genetic inhibition of TNF could attenuates renal fibrosis by reducing macrophage infiltration and pro-fibrotic mediators (Zhang et al. 2023). Therefore, targeting the inhibition of TLR or TNF signaling pathway could be seen as a novel therapeutic strategy to attenuate renal fibrosis.

G-T-P-M network analysis revealed that Propionate, Butyrate, 3-Indolepropionic acid and Trimethylamine oxide were recognized as the top 4 metabolites. Propionate and butyrate are categorized as SCFAs (Tillett et al. 2025). Studies have found that reduced fecal concentrations of propionate and butyrate in patients with chronic kidney disease (CKD) are strongly correlated with decreased renal function accopmanied by elevated Scr and BUN levels (He et al. 2024). In a folic acid-induced AKI-to-CKD mouse model, administration of propionate or butyrate could reduce the production of ECM and decrease the expression of renal injury markers KIM-1 and NGAL (Wang et al. 2024). Mechanistically, the renal protective mechanisms of propionate or butyrate mainly involve the following aspects: propionate or butyrate inhibits histone deacetylase (HDAC) and activates G protein-coupled receptors (GPR43/GPR109A), blocks the TLR2-mediated NF-κB/p38 MAPK signaling pathway, and reduces the release of pro-inflammatory and fibrotic factors (Corte-Iglesias et al. 2024). Meanwhile, propionate or butyrate also exert renal protection by regulation of immune response through the inhibiting recruitment of neutrophils and monocytes, regulating the phenotypic transformation of macrophages to the anti-inflammatory M2 type (Mann et al. 2024). Additionally, propionate or butyrate inhibits epithelial-mesenchymal transition (EMT) and promotes renal tubular repair through epigenetic regulation (Corte-Iglesias et al. 2024). 3-Indolepropionic acid (IPA), a natural product of tryptophan metabolism by gut microbiota, has demonstrated potential clinical value in diseases such as chronic kidney disease and cancer (Guijas et al. 2022). However, its role in liver fibrosis remains controversial (Yuan et al. 2023): in a diet-induced non-alcoholic steatohepatitis (NASH) rat model, IPA suppressed the expression levels of liver fibrosis-related genes and alleviates fibrosis severity; conversely, the administration of IPA exacerbated liver fibrosis in a mouse model of liver injury. This contradiction may be attributed to the modulating effects of factors including diet, genetic background, and intestinal microenvironment on the gut-liver axis. The chronic exposure to high levels of Trimethylamine oxide is closely link to renal fibrosis (Gatarek and Kaluzna-Czaplinska 2021). Animal experiments indicated that the pro-fibrotic effects of TMAO are mainly mediated through multiple signaling pathways. Trimethylamine oxide could activate the TGF-β/SMAD3 pathway, which served as key regulator in renal fibrosis by promoting extracellular matrix deposition (Gungor et al. 2024). The NLRP3 mediated inflammasome could be activated by TMAO that induce the overexpression of inflammatory factors such as IL-1β and IL-6. The aggravated inflammatory response further exacerbated tissue jury and fibrosis (Bai et al. 2024). Therefore, the inhibition of TMAO could serve as an anti-fibrotic therapeutic strategy by reducing oxidative stress and endoplasmic reticulum stress.

Based on network pharmacology research, our study demonstrated that the identified targets and metabolites through data mining provide a new perspective on the potential therapeutic effects of gut microbiota metabolites on renal fibrosis. However, there are still some limitations in this study. Firstly, the study mainly relies on computational analyses of network pharmacology and molecular docking, lacking animal or cellular experiments to validate the actual effects of the identified targets and metabolites in renal fibrosis, which may lead to limitations in mechanism interpretation. Secondly, the data used in this study are acquired from public databases, which may have incomplete coverage or annotation biases for gut microbiota metabolite related targets and renal fibrosis-related genes, affecting the accuracy of target and pathway screening. What’s more, the study does not take the influence of individual differences in gut microbiota composition on metabolite production into consideration. In addition, other interfering factors such as host genetic background may influence the regulatory network of the “gut-kidney axis” that limit the application of research results to clinical translation.

## Conclusion

In conclusion, our study integrating network pharmacology and bioinformatics represents a promising assessment method that offers prospective insights into complex interactions and systems biology between gut microbiota metabolites and renal fibrosis. This integrative analytical approach not only expands the knowledge of gut microbiota metabolites’ therapeutic mechanisms but also open a novel avenue for exploring complex biological processes in the context of renal fibrosis.

## Data Availability

The data are available upon request.

## Abbreviations

PPI: Protein protein interaction network
GO: Gene ontology
KEGG: Kyoto encyclopedia of genes and genomes
SMILES: Simplified molecular input line entry system
PAMPs: Pathogen-associated molecular patterns
DAMPs: Damage-associated molecular patterns
EMT: Epithelial-to-mesenchymal transition
SCFAs: Short-chain fatty acids
CKD: chronic kidney diseases
TNF: tumor necrosis factor
TLR: Toll-like receptor
AKT1: AKT serine/threonine kinase 1
IL6: Interleukin 6
IL1B: Interleukin 1β
